# Recent Advances of Exosomes Derived from Skeletal Muscle and Crosstalk with Other Tissues

**DOI:** 10.3390/ijms252010877

**Published:** 2024-10-10

**Authors:** Jia Luo, Qiang Pu, Xiaoqian Wu

**Affiliations:** 1 College of Animal Science and Technology, Southwest University, Chongqing 400715, China; 2 College of Food Science, Southwest University, Chongqing 400715, China

**Keywords:** skeletal muscle, exosome, crosstalk, adipose, bone

## Abstract

Skeletal muscle plays a crucial role in movement, metabolism, and energy homeostasis. As the most metabolically active endocrine organ in the body, it has recently attracted widespread attention. Skeletal muscle possesses the ability to release adipocytokines, bioactive peptides, small molecular metabolites, nucleotides, and other myogenic cell factors; some of which have been shown to be encapsulated within small vesicles, particularly exosomes. These skeletal muscle exosomes (SKM-Exos) are released into the bloodstream and subsequently interact with receptor cell membranes to modulate the physiological and pathological characteristics of various tissues. Therefore, SKM-Exos may facilitate diverse interactions between skeletal muscle and other tissues while also serving as biomarkers that reflect the physiological and pathological states of muscle function. This review delves into the pivotal role and intricate molecular mechanisms of SKM-Exos and its derived miRNAs in the maturation and rejuvenation of skeletal muscle, along with their intercellular signaling dynamics and physiological significance in interfacing with other tissues.

## 1. Introduction

Skeletal muscle, constituting 40–60% of body weight, serves as the largest organ in the human body and plays a crucial role in movement, systemic metabolism, energy homeostasis; and it also serves as the primary source of animal protein for humans. The organism functions as a complex network of interactions, with extensive information exchange occurring between cells. Living cells are capable of responding to external signals, thereby influencing their own processes of division, differentiation, and function. The skeletal muscle consists of a diverse range of cell types, including muscle cells, stem cells, fibroblasts, and immune cells et al. [1]. The intricate intercellular communication among these cell populations is essential for maintaining skeletal muscle homeostasis and function [2]. Over the past two decades, skeletal muscle has gradually been recognized as a secretory organ that releases various humoral factors known as myokines, which regulate the homeostasis and adaptation of peripheral organs, thus influencing overall body homeostasis [3,4]. Metabolomics analyses reveal that many secretory factors of skeletal muscle are released into the bloodstream to communicate with other organs. These factors exist in free or protein-bound forms, and some are packaged in exosomes, which regulate target cells [5].

Exosomes derived from skeletal muscle (SKM-Exos) and muscle factors are effective mediators of skeletal muscle cell differentiation, proliferation, and metabolism, thereby creating a microenvironment for intercellular interaction within different groups of skeletal muscle cells [6,7]. They are essential contributors to the regulation of muscle physiology and whole-body homeostasis, playing a pivotal role in the physiology of muscles and systemic homeostasis through paracrine signaling. Additionally, exosomes play a pivotal role in regulating cell fate and facilitating tissue regeneration [8]. It has been demonstrated that SKM-Exos releases into the extracellular environment, enhancing our understanding of how skeletal muscle communicates with other tissues [4]. Exosomes confer distinct properties based on their organ and tissue of origin, leading to a bias towards specific organs or uptake by particular cell types [9]. In fact, over 1000 RNAs have been identified in exosomes along with proteins/peptides, lipids, and metabolites [10]. Through this process, secreted exosomes engage with the cell membrane of target cells to mediate intercellular and interorgan communication. Among the various cargoes transported by exosomes, miRNA stands out as the most significant signaling molecule, exerting profound effects on diverse cellular functions through transcriptional and post-transcriptional regulation. Its involvement is critical in numerous physiological and pathological processes [11,12]. SKM-Exos have been demonstrated to be highly concentrated in the skeletal muscle interstitium, containing a high enrichment of muscle-specific miRNAs, and capable of inhibiting the expression of Pax7, a key regulator of muscle production [13]. Additionally, SKM-Exos secrete age-related miRNAs that are elevated in exosomes released by skeletal muscle myocytes and fibroblast progenitors. These miRNAs have the potential to promote cartilage degeneration and impact neighboring hematopoietic stem cells as well as circulate in the blood [14]. Exosomes derived from muscle tissue are significantly associated with muscle atrophy. For instance, exosomes isolated from the culture medium of inflammatory myoblasts (C2C12) induce muscle atrophy by promoting inflammation in myoblasts and inhibiting their differentiation process [15]. BMSC-derived exosomes inhibit dexamethasone-induced muscle atrophy via miR486-5p/Foxo1 Axis [16]. This underscores the therapeutic potential of exosome-based interventions for Duchenne muscular dystrophy (DMD) and other skeletal muscle disorders characterized by compromised function. Numerous studies have demonstrated that SKM-Exos serve a dual role in paracrine and endocrine signaling, crucial for maintaining muscle homeostasis and facilitating communication with other tissues [5,17]. Myokines and exosomes originating from skeletal muscle are known to significantly contribute to the maintenance of brain homeostasis. Myoblasts and myotubules represent two types of myocytes that are sources of exosomes expressing protein markers such as the quadritransmembrane protein family, tumor susceptibility gene 101 (TSGl01), apoptotic link-factor 2-interaction protein x (Alix), along with other proteins involved in signal transduction [4,18]. The released exosomes can transport their proteins, mRNA, and miRNA to recipient cells, thereby regulating target cell functions and mediating intercellular communication as well as inter-tissue interactions. Skeletal muscle-derived exosomes play a pivotal role in the proliferation, differentiation, and repair of myoblasts. In light of this, we postulate that the interplay between SKM-Exos and cells plays a pivotal role in muscle development, regeneration, and crosstalk with other tissues and organs, potentially modulated by miRNAs. Consequently, this manuscript offers a systematic synthesis of these fields and presents a comprehensive overview of the advancements in research.

## 2. The Characterization of Exosomes Derived from Skeletal Muscle

Guescini et al. first demonstrated that skeletal muscle cells can produce exosomes in 2010 [18]. SKM-Exos contains myofactors and other biological regulators, such as esRNA, cytokines, chemokines, and prostaglandins, which may also modulate the remodeling of other important sites. Hence, SKM-Exos has gained increasing recognition for its advantageous roles in the regulation of metabolism, cellular differentiation, and tissue regeneration in recent years. SKM-Exos play a role in cellular communication and protect biomolecules during transportation in biological fluids. During this process, the exosomes are also crucial for the stability of cytokines [19]. The SKM-Exos are capable of transporting miRNA, mRNA, lncRNA, proteins, lipids, and other molecules released by viable cells and delivering them to target cells through diverse mechanisms, thereby inducing cellular responses. This establishes its significance as a crucial mediators in intercellular communication [2,20]. The contents released by SKM-Exos have biological effects on adjacent or distant cells [20]. The exosome-wrapped miRNAs regulate pathways related to muscle quality control, calcium signaling, and neuromuscular junction function [21,22,23]. SKM-Exos contain four myomiRs (miR-1, miR-133a, miR-133b, and miR-206), some of which enter the bloodstream while others are utilized for local communication between nearby muscle tissues to influence their own fate and biological activities, such as muscle remodeling [24,25]. For instance, myogenic progenitor cells (MPCs) contribute to muscle remodeling in response to hypertrophic stimuli through exosome miR-206 [26]. However, exosomes from inflammatory mouse myoblasts’ conditioned media contribute to induced myoblast inflammation and inhibited myogenic mechanisms while stimulating atrophic signals [15]. In addition to inflammatory models, myotube-derived exosomes improved muscle function and alleviated degeneration in malnourished mice by enhancing muscular membrane integrity, demonstrating the therapeutic potential of exosomes for Duchenne muscular dystrophy (DMD) and other skeletal muscle diseases with damaged membranes [27]. In summary, the content of SKM-Exos, such as miRNAs, changes in response to both physiological and pathological stimuli, subsequently influencing the fate of skeletal muscle. Hence, it can be employed in investigating the molecular mechanisms underlying skeletal muscle development and in addressing skeletal muscle disorders.

Exosome-wrapped proteins and peptides are also important for cell signaling, membrane vesicle transport, and migration. In addition to miRNAs, proteins, and lipids in SKM-Exos play a crucial role in various physiological and pathophysiological processes. For instance, the proteins carried by SKM-Exos can promote muscle cell fusion and muscle growth [25]. A lipidomics analysis indicates that SKM-Exos can transfer lipids between muscle cells. Palmitic acid-induced skeletal muscle insulin resistance promotes the release of SKM-Exos enriched in palmitic acid. The release of SKM-derived exosomes can mitigate the accumulation of fatty acids within cells. Lipids constitute a crucial component of the exosomal membrane, with specific lipids being enriched in these vesicles [28,29]. This enrichment, along with a high protein-to-lipid ratio and asymmetric distribution, is associated with increased membrane rigidity. Muscle-derived exosomes are particularly rich in palmitic acid, stearic acid, oleic acid, palmitoleic acid, and lauric acid-fatty acids that serve as energy sources or contribute to the formation of phospholipid bilayers in cell membranes [30]. Palmitate-cultured C2C12 cell-derived exosomes induced myoblast proliferation and modified the expressions of genes involved in the cell cycle and muscle differentiation. Skeletal muscle could transfer specific signals through the exosomal route to key metabolic tissues, and exosomes can modify muscle homeostasis during high-fat diets [30]. Moreover, SKM-Exos could be secreted into extracellular fluids and migrate to the skeletal muscle cells as well as other organs and regulate metabolic and immunological functions [5]. Therefore, thoroughly investigating the interaction and communication between SKM-Exos and other tissues constitutes an indispensable aspect of comprehensively studying the overall internal environmental homeostasis.

## 3. The Role of SKM-Exos in Muscle Development and Regeneration

The process of mammalian skeletal myogenesis is intricate, enabling precise regulation of the proliferation, differentiation, and fusion of myogenic cells to generate multinucleated, contractile, and functional muscle fibers. Precise cellular proliferation and differentiation control are vital for proper embryonic and post-embryonic skeletal muscle development [31]. Seletal muscle develops from the lateral mesodermal side of myoblast cells, which undergo terminal differentiation to form mononuclear myoblast cells. These cells then migrate to the site of muscle formation and go through a series of processes, including proliferation, differentiation, and fusion, to ultimately generate functional muscle tissue [31,32]. After birth, muscle fibers remain largely unchanged in composition, primarily growing through hypertrophy and regeneration by satellite cells. In cases of injury, satellite cells can be activated to facilitate muscle repair by re-entering the proliferation and differentiation process. Therefore, precise regulation of cell proliferation and differentiation is crucial for normal fetal and postnatal muscle development [33,34]. Emerging studies have found that SKM-Exos can enhance the proliferation and differentiation of skeletal muscle cells, as well as regulate the expression of muscle-related genes. Incubation of myoblasts with H_2_O_2_-processed myotubes exosomes resulted in a significant reduction in myogenin (MyoG) levels and myosin heavy chain (MHC) expression, accompanied by an upregulation of proliferating cell nuclear antigen (PCNA). These combined effects ultimately led to an enhancement in recipient myoblast proliferation [35]. Under physiological conditions, miRNAs, proteins, and muscle cytokines in SKM-Exos regulate the proliferation, differentiation, and fusion of skeletal muscle cells. These molecules also have the potential to modulate or improve pathological processes. For instance, miR-130a promotes cell proliferation, while miR-133b inhibits tumor growth [20]. According to previous research findings, the miRNAs present in SKM-Exos play a multifaceted role in the regulation of muscle cell proliferation and differentiation. Intact skeletal muscle tissues release exosomes containing the four myomiRs, namely miR-1, miR-133a, miR-133b, and miR-206; significantly elevated levels of these were detected in the cytoplasm and exosomes of mouse myoblasts as well as human skeletal muscle cells during their proliferation and differentiation [36]. Exosomes secreted during myotube differentiation fused with neighboring cells at early time points and exhibited a myotube-like phenotype with increased expression of myogenic proteins [37]. Conversely, in pathological conditions, myogenic exosomes inhibit the differentiation of myoblasts and induce muscle atrophy [15]. These studies suggest that the variations in the regulatory functions of SKM-Exos on myoblast differentiation are associated with the distinct components of exosomes secreted under different physiological conditions.

SKM-Exos can promote myogenesis by delivering miRNAs between proliferating myoblasts and maturing myotubes [25]. In this context, exosomal miRNAs released by myotubes can suppress Sirt1 in myoblasts, contributing to myoblast commitment during differentiation. Importantly, the specific miRNA subsets released by myotube-derived exosomes differ from those derived from C2C12 cells [38]. The diverse microenvironments surrounding SKM-Exos may contribute to the discrepancies observed in various studies. Research has demonstrated that myoblasts release exosomes rich in miRNAs within an inflammatory environment, facilitating the transfer of miR-224 into macrophages to inhibit M2 polarization. As a result, the secretome of M1 macrophages impedes myogenic differentiation while promoting proliferation. Exosomes derived from inflamed myoblasts promote M1 polarization and hinder myoblast differentiation, leading to increased myogenic proliferation and disrupting the balance between myoblast proliferation and differentiation [39]. Therefore, the interplay between myoblasts and macrophages via miRNA-containing exosomes during muscle healing has important implications for regulating macrophage polarization as well as myoblastic differentiation and proliferation [40].

In addition to miRNAs, proteins and lipids in SKM-Exos also play crucial roles in various physiological and pathophysiological processes. The proteins involved in the formation of myoblast-sarcoplasmic-tubules in SKM-Exos exhibit similar biological functions to miRNAs, while those carried by SKM-Exos can facilitate muscle cell fusion and growth. For example, exosomes derived from C2C12 cells contain ITGB1, CD9, CD81, NCAM, and myosin, which promote myoblast recognition and adhesion for facilitating fusion [38]. Furthermore, SKM-Exos facilitate lipid transfer between muscle cells. Normally, fatty acids produced by skeletal muscle are used for energy or to form phospholipid bilayers. However, excessive intracellular accumulation of fatty acids is associated with insulin resistance and glucose metabolism disorders [30]. SKM-Exos can be internalized by pancreatic beta cells and deliver functional cargoes, such as miRNAs, potentially exerting endocrine effects and contributing to adaptations in beta cell mass during insulin resistance [41]. MiR-146a-5p derived from SKM-Exos can suppress lipid droplet formation and preadipocyte differentiation by downregulating GDF5 expression and inhibiting the PPARγ signaling pathway. In mice, specific knockout of miR-146a-5p leads to a significant increase in body weight and a reduction in oxidative metabolism levels, accompanied by a notable rise in lipogenesis [42]. Therefore, exosome release represents a cellular self-protection mechanism against intracellular fatty acid accumulation.

When skeletal muscle is injured, satellite cells are activated and rapidly proliferate, fusing with damaged muscle fibers to form new muscle fibers, promoting muscle growth and tissue repair. The promising effects of exosomes on promoting satellite cell differentiation and upregulating myogenesis genes in injured skeletal muscle have been established thoroughly in several studies. MSC-derived exosomes promote muscle regeneration by enhancing myogenesis and angiogenesis, which is at least in part mediated by miRNAs such as miR-494 [21]. Exosomes released by muscle cells can initiate the myogenic process in satellite cells and provide biochemical signals for skeletal muscle regeneration [22,43]. Studies have demonstrated that exosomes derived from myoblasts are involved in multiple biological processes and can enhance the regeneration of skeletal muscle fibers through the modulation of satellite cell proliferation, fibroblast-like cell differentiation, and regulation of satellite cell mRNA expression and protein turnover [43]. In previous studies, SKM-Exos derived from the muscle tubules could significantly improve the integrity of the muscle tubule membrane and reduce the influx of calcium into the cell, suggesting that extracellular vesicles may have beneficial effects in slowing down the pathological progression of muscular dystrophy in mouse models [27]. In addition, used as an engineered exosome vector, exosome-encapsulated miR-29 ameliorates skeletal muscle atrophy and attenuates kidney fibrosis by downregulating YY1 and TGF-β pathway proteins. Injecting miR-29-carrying exosomes into the gastrocnemius muscle can prevent muscle atrophy induced by chronic kidney disease [44]. These results suggest that targeting SKM-Exos could be a promising therapeutic approach for the prevention and treatment of muscle atrophy.

However, exosomes released under injury or atrophic pathological states differ from those under physiological conditions. For instance, the alterations in miR-23a following muscle atrophy validate the involvement of muscle-derived exosomal miRNAs in muscle atrophy and their contribution to pathological progression [45]. Exosomes produced by local fibroblasts in the Duchenne muscular dystrophy (DMD) muscle are able to induce phenotypic conversion of normal fibroblasts to myofibroblasts, thereby increasing the fibrotic response. This conversion is related to the transfer of high levels of miR-199a-5p and to the reduction in its target caveolin-1 [46]. Hence, a comprehensive investigation into the heterogeneity of SKM-Exos function across diverse physiological contexts can offer valuable insights for its efficacious application in addressing skeletal muscle injuries and atrophy in future therapeutic interventions.

## 4. Crosstalk between SKM-Exos and Other Tissues

In addition to acting on target cells through releasing SKM-Exos, the muscle cells act as target cells and are regulated by exosomes released from other cells. For instance, miR-27a in adipocyte-derived exosomes contributes to insulin resistance in skeletal muscle by targeting PPARγ [47]. Previous studies have shown that skeletal muscle cells can produce exosomes both in vitro and in vivo, with SKM-Exos reaching the circulation [1,2,48]; These SKM-Exos travel through the bloodstream to reach other tissues, including adipose tissue and bone [49,50,51]. The following section summarizes the role and progress of SKM-Exos in interacting with other tissues.

### 4.1. Muscle-to- Adipose Crosstalk

SKM-Exos can communicate with various tissues and transmit specific signals to key metabolic tissues [12,30]. Despite their distinct structures and functions, muscle, and adipose tissue located in different parts of the body are closely interconnected. Additionally, both tissues act as endocrine organs by releasing cytokines that play a crucial role in regulating tissue balance under various conditions [52]. Understanding the complex interaction between muscle and adipose tissue has always been a key focus of research. In skeletal muscle, muscle cells are closely linked to neighboring adipocytes through the exchange of cytokines and exosomes. Moreover, these molecules released by skeletal muscle can influence whole-body energy metabolism via systemic circulation, affecting processes such as differentiation, regeneration, glucose homeostasis, and fatty acid balance. Recent findings indicate that exosomes derived from muscle can impact the phenotype and stability of nearby recipient cells, with SKM-Exos playing a crucial role in adipose tissue development [19]. While the regulatory mechanisms of classical myogenic factors in the muscle-adipose axis have been extensively investigated, recent studies have unveiled the involvement of SKM-Exos in mediating muscle-adipose interactions and modulating whole-body metabolism [19]. Experimental evidence demonstrates the release of SKM-Exos from muscle fibers or into the conditioned medium of skeletal muscle cells. In recent years, numerous SKM-Exos miRNAs have been identified by researchers as valuable biomarkers and potential targets for the treatment of various diseases, and they also mediate muscle-adipose crosstalk [42,47,53]. Treatment of adipocytes with SKM-Exos leads to down-regulation of target proteins (Smarcd1 and Runx2) by miR-133a, suggesting that miRNAs in SKM-Exos may be transferred to precursor adipocytes to regulate gene expression. This provides initial evidence that SKM-Exos may signal nearby adipocytes through paracrine signaling [54]. Under healthy conditions, SKM-Exos serve as an anti-lipogenesis signal by inhibiting lipid accumulation in 3T3-L1 adipocytes and differentiation of fiber/adipose-derived progenitors into adipocytes. For example, SKM-Exos facilitate the metabolic adaptation of adipose tissue to mechanical overload by mediating miR-1 transmission [55]. Notably, SKM-Exos significantly suppress preadipocyte differentiation and adipogenesis, while miR-146a-5p plays a crucial role in regulating adipogenesis and obesity through the modulation of the skeletal muscle-fat signaling axis. Co-treatment with SKM-Exos alongside miR-146a-5p inhibitors reversed this inhibitory effect, indicating its essential role in maintaining a balance between normal skeletal muscle development and adipogenesis [42]. These studies demonstrate that SKM-Exos enriched with miRNAs play a crucial regulatory role in differentiation and development processes within adipocytes. Additionally, SKM-Exos can also exert localized effects on adipose tissue development, enhance the lipidogenic activity of intramuscular adipocytes, and demonstrate heterogeneity within skeletal muscle. There are fundamental distinctions between the myogenic exosome-mediated muscle adipose tissue crosstalk in fast and slow muscles. For instance, SKM-Exos derived from slow muscles promote lipid accumulation in intramuscular adipocytes, whereas SKM-Exos derived from fast muscles inhibit lipid accumulation [1]. Numerous studies have reported the presence of exosomes and miRNAs originating from skeletal muscle involved in intercellular communication between muscle and adipose cells. However, several unresolved issues remain, particularly regarding the mechanisms underlying their contribution to whole-body metabolic regulation, necessitating further investigation. Furthermore, how do exosomes released by different muscle fibers participate in the muscle-fat interaction? Do they also regulate lipid balance in adipose cells across different types of adipose tissue? Therefore, a systematic study of SKM-Exos heterogeneity and its regulatory mechanism on adipose tissue development is particularly important for understanding the balance between muscle and adipose tissue interaction.

### 4.2. Muscle-to-Bone Crosstalk

As integral components of the musculoskeletal system, skeletal muscle and bone typically exhibit coordinated development and growth. Therefore, elucidating the molecular crosstalk between skeletal muscle and bone holds promise for advancing our understanding of conditions such as sarcopenia and osteoporosis. It was previously widely believed that the mechanical load resulting from muscle contraction constituted the primary mechanism through which muscle influenced bone density [56]. Subsequently, it was discovered that muscle tissue has the capacity to act as an endocrine pathway by releasing ‘myogenic factors’ [57]. There is mounting evidence supporting the cross-communication of signals between bone and skeletal muscle via circulatory and local mediators, including growth hormone IGF-1 and FGF-2, osteocalcin, and irisin, which are believed to functionally link muscle and bone [58,59,60,61,62]. Do exosomes, particularly SKM-Exos, also serve as mediators of musculoskeletal intercellular communication? There is growing evidence that skeletal muscle, in addition to being the largest tissue responsible for movement, also functions as an important endocrine tissue regulating metabolism in the body [63]. Exosome-mediated intercellular signaling plays a critical role in maintaining the stability and functionality of muscle and bone tissues [64]. SKM-Exos can be taken up by other organs, where they may exert functional effects. For instance, fluorescence-labeled SKM-Exos were administered via the tail vein of mice and observed to permeate most tissues within 24 h, including the lungs, liver, spleen, brain, heart, pancreas, and gastrointestinal tract. SKM-Exos can circulate in the bloodstream and target the bone, where they are internalized by bone marrow mesenchymal stem cells (BMSCs), thereby enhancing glycolysis in BMSCs through the delivery of lactate dehydrogenase A. SKM-Exos facilitate osteogenic differentiation of BMSCs and confer protective effects against disuse osteoporosis in mice [49]. With the progressive aging of skeletal muscle, the bioactive substances present in exosomes secreted by skeletal muscle cells exhibit significant variations compared to those secreted by young skeletal muscle cells, consequently leading to altered effects on bone metabolism [14,50]. Compared to young SKM-Exos, aged SKM-Exos may disrupt the dynamic balance of bone metabolism by inhibiting the osteogenic differentiation of primary osteoblasts, promoting the formation of osteoclasts, and leading to osteoporosis [65]. Exosomes secreted by myoblasts can be internalized by osteoblasts, contingent upon the release of miR-27a-3p from these exosomes and the subsequent activation of the β-catenin signaling pathway, which promotes osteoblast differentiation. It was observed that the expression of miR-34a was significantly upregulated in exosomes derived from aging skeletal muscle, and overexpression of miR-34a in C2C12 exosomes markedly decreased the viability of bone marrow mesenchymal cells and impacted bone metabolism [66,67]. Hence, exosomes derived from aging skeletal muscle have the potential to promote bone aging through miRNA modulation. Exosomal miRNAs can be considered a novel class of ‘myokines’ with the potential to promote osteogenesis, thereby enhancing our understanding of muscle-bone interactions under both physiological and pathological conditions. Aged skeletal muscle generates circulating SKM-Exos that can induce senescence in stem cell populations within the bone and other tissues through their miRNA cargo [66,67]. These findings suggest that circulating muscle-related miRNAs may serve as biomarkers for evaluating bone disease states and could potentially reflect responses to therapeutic interventions.

## 5. Conclusions

At present, extensive research has been conducted on SKM-Exos in the context of skeletal muscle development, proliferation, differentiation, and regeneration of enveloped myoblasts. However, there is still a lack of understanding regarding the communication between SKM-Exos and other tissues, as well as their role in regulating body homeostasis. Specifically, further investigation is needed to explore the involvement of SKM-Exos in adipose tissue homeostasis and bone aging. Current studies on the interaction between skeletal muscle and other tissues primarily focus on unidirectional signaling actions; therefore, it is important to investigate whether there exists a mutual balance between them. Additionally, it has been observed that exosomes from different skeletal muscles exhibit distinct biological functions. Hence, exploring the heterogeneity of skeletal muscle exosomes and its potential involvement in cellular “dialogue” is an important area for further discussion within the study of skeletal muscle development and its interaction with other tissues. Moreover, the potential use of SKM-Exos as biomarkers for sarcopenia, aging and various diseases remains unclear. Consequently, one of the main challenges anticipated in the coming years will be to enhance the efficiency of translating findings from animal physiology to human physiology and subsequently into medical applications.

## Data Availability

No new data were created or analyzed in this study.
